# Down-Regulation of *MiR-181c-5p* Promotes Epithelial-to-Mesenchymal Transition in Laryngeal Squamous Cell Carcinoma *via* Targeting *SERPINE1*

**DOI:** 10.3389/fonc.2020.544476

**Published:** 2020-12-21

**Authors:** Xin Li, Ping Wu, Yaoyun Tang, Yuhua Fan, Yalan Liu, Xing Fang, Wei Wang, Suping Zhao

**Affiliations:** ^1^Department of Otorhinolaryngology Head and Neck Surgery, Xiangya Hospital of Central South University, Changsha, China; ^2^Province Key Laboratory of Otolaryngology Critical Diseases, Xiangya Hospital of Central South University, Changsha, China; ^3^National Clinical Research Center for Geriatric Disorders, Xiangya Hospital of Central South University, Changsha, China

**Keywords:** miRNA, mRNA, laryngeal squamous cell carcinoma, epithelial-to-mesenchymal transition, biomarker

## Abstract

Laryngeal squamous cell carcinoma (LSCC) arises from the squamous epithelium of the larynx and is associated with a high incidence of cervical lymph node metastasis. MicroRNAs (miRNAs) play a crucial role in the epigenetic regulation of cellular biological processes, including cancer metastasis. However, the molecular mechanisms of specific miRNAs responsible for LSCC metastasis and their clinical significance have yet to be fully elucidated. In this study, LSCC cohort datasets from the Gene Expression Omnibus (GEO) and The Cancer Genome Atlas (TCGA) were downloaded and examined by comprehensive bioinformatics analysis, which revealed that upregulation of mRNA *SERPINE1* and downregulation of *miR-181c-5p* were associated with unfavorable overall survival. Our analysis showed that *SERPINE1* expression negatively correlated with the expression level of *miR-181c-5p* in our LSCC patient samples. Silencing of *miR-181c-5p* expression promoted cell migration and invasion in cell lines, whereas the overexpression of *miR-181c-5p* suppressed cell migration and epithelial-to-mesenchymal transition (EMT) through the downregulation of *SERPINE1*. Further analysis showed that the enhancement effect on EMT and metastasis induced by silencing *miR-181c-5p* could be rescued through knockdown of *SERPINE1* expression *in vitro*. Collectively, our findings indicated that *miR-181c-5p* acted as an EMT suppressor miRNA by downregulation of *SERPINE1* in LSCC and offers novel strategies for the prevention of metastasis in LSCC.

## Introduction

Laryngeal cancer (LC) is the most common malignant neoplasm in head and neck. The predominant histopathological characteristic of LC is squamous cell cancer; this can affect different regions of the larynx and is associated with different symptoms and various treatment approaches (1, 2). Epidemiologically, the incidence of laryngeal squamous cell cancer (LSCC) has increased over recent years and now represents a serious threat for human health. Each year, over 110,000 new cases are diagnosed and of these, approximately 40 percent progress to an advanced stage (3). Tobacco smoking and alcohol consumption are known to be significant risk factors for LSCC (4). Therapeutic interventions for patients with LSCC vary according to the stage of cancer, and can involve surgery, radiotherapy, adjuvant radiation, or chemoradiation, which can be applied as a single modality or as a multimodal strategy (2). However, due to loco-regional relapse and/or distant metastasis, the overall survival (OS) of advanced LSCC has improved only slightly over the past decades and appropriate treatment remains a major challenge. The identification of molecular biomarkers for LSCC metastasis could help to improve the prognosis of patients and may also facilitate the prediction of patient survival.

MicroRNAs (miRNAs) are a class of small non-coding single-stranded RNA molecules that are located on the endogenous chromosomes. These molecules are approximately 19–25 nucleotides in length and are known to play a role in post-transcriptional gene regulation (5). MiRNAs can regulate the expression of target genes involved in the development and progression of cancer by acting as oncogenes or tumor suppressor genes (6). Over recent years, evidence has grown to support the potential regulatory role of miRNAs in the pathological changes that occur in LSCC. For example, *mir-141*, *mir-203*, and *miR-1469* can influence the progression of LSCC cells *via* their involvement in epithelial-to-mesenchymal transition (EMT) (7), lymph node metastasis (8), and the p53-mediated pathway (9). Other studies have indicated that *miR-21*, *miR-155*, *miR-192*, and *miR-375* regulate activation of the NF-kB pathway in LSCC (10). Therefore, these specific miRNAs may have great potential as LSCC biomarkers to improve the early detection.

EMT is a complex process through which epithelial cells lose their characteristic features such as cell polarity and cell–cell adhesion, and gain a mesenchymal-like phenotype including the acquisition of migratory and invasive properties (11). Carcinoma cells in primary tumors are able to reactivate the EMT program to promote new invasive and metastatic properties (12). Genetic and epigenetic programs are involved in the regulation of EMT transcription factors (EMT-TF), including, but not limited to, SNAI1/2, TWIST1, and ZEB1/2, which can repress epithelial (*e.g.*, E-cadherin) or activate the transcription of mesenchymal markers (*e.g.*, vimentin, fibronectin, N-cadherin) (13). Thus, the activation status of EMT can be evaluated through the detection of the dynamic changes of these protein markers. Recent research has demonstrated that miRNAs play a critical role in metastasis and recurrence, that is, by regulating genes involved in EMT, migration, and invasion (14). For example, upregulation of miR-21 has been reported to be correlated with lymph node metastasis in head and neck squamous cell carcinoma (HNSCC) and actives the expression of cyclin-dependent kinase 5 through the STAT3/miRNA-21 pathway, which has also been shown to promote EMT (15). Conversely, miR-26a/b, miR-29a/b/c, and miR-218 by regulating LOXL2 expression have been reported to inhibit cancer cell migration and invasion in HNSCC (16). However, the role and mechanism of specific miRNAs involved in EMT and their influence on cell invasion and metastasis in LSCC, are still not well understood.

**Figure 1:**
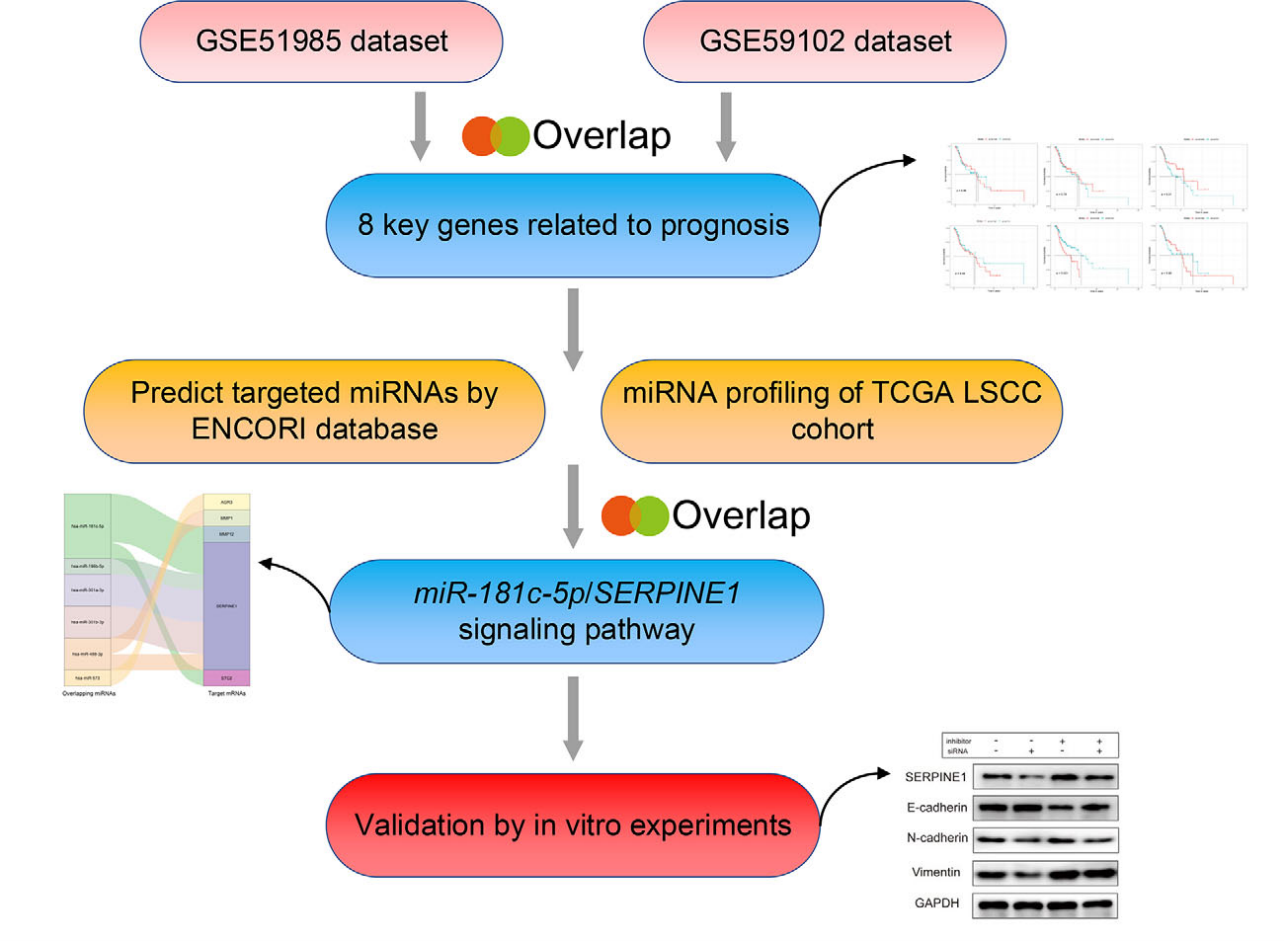
The bioinformatics analysis strategy in this study.

Herein, we designed an experimental strategy to identify crucial miRNAs, and their relative target genes, involved in metastasis and invasion of LSCC (**Figure 1**). First, the expression profiles of mRNAs and miRNAs in LSCC samples were downloaded from the Gene Expression Omnibus (GEO) (GSE51985, GSE59102) and The Cancer Genome Atlas (TCGA) databases. A comprehensive series of analyses subsequently identified *miR-181c-5p/SERPINE1* as being dysregulated in LSCC. In addition, functional experiments *in vitro* demonstrated that *miR-181c-5p* negatively regulated *SERPINE1*, a potential oncogene, and significantly repressed the invasion, metastasis, and EMT of LSCC. In conclusion, our findings indicated that *miR-181c-5p* could potentially be used as a novel biomarker and therapeutic target in LSCC patients.

## Materials and Methods

### Raw Data Acquisition and Identification of Differentially Expressed mRNAs

Two expression profiling datasets (GSE51985, GSE59102) were downloaded from the GEO database at the NCBI using the following search terms: ((“gene” OR “mRNA” OR “miRNA” [all fields] AND (“laryngeal cancer” OR “laryngeal squamous cell carcinoma” [all fields]) AND “Homo sapiens” [porgn]). The GSE51985 and GSE59102 datasets, based on the Illumina HumanHT-12 V4.0 expression BeadChip platform, and the Agilent-014850 Whole Human Genome Microarray 4x44K G4112F platform, respectively contained a total of 62 tissue samples (39 LSCC tissues and 23 adjacent cancer tissues). In addition, the TCGA LSCC cohort was used to obtain differentially expressed (DE) miRNAs and contained 117 LSCC tissues and 12 normal tissues.

Original gene expression profiles were obtained from the two mRNA datasets. Differently expressed mRNAs (DE mRNAs) were identified by GEO2R, using the “limma” package in R; therefore secondary normalization was unnecessary. Volcano plots of the mRNAs contained in the two GEO datasets were built using the ggplot2 package in Ra. The log2 fold change (FC) was set as the abscissa and the negative logarithm of the P-value as the ordinate. The threshold of the DE mRNAs in both datasets was set as log2 (FC) >2 or < −2 and P <0.05. Venn diagrams were used to identify overlapping genes and were created by the set of online tool: Venny 2.1 (17). We also used the “pheatmap” package in *R* to create a heatmap. DE miRNAs were identified from the above cohorts using a threshold of log2 (FC) >2 or <−2 and P<0.05.

### Enrichment Analysis and PPI Network Construction

In order to demonstrate functional annotations, we used the “clusterProfiler” package in *R* to perform Gene Ontology (GO) functional annotation and the Kyoto Encyclopedia of Genes and Genomes (KEGG) pathway enrichment analysis (18). GO functional annotations were sorted into three broad categories: molecular function (MF), biological process (BP), and cellular components (CC). Using these three functional categories, gene function could be defined and described from multiple aspects. Ultimately, the significant terms or pathways were displayed using the “ggplot2” package in R. To analyze the connection between proteins and to score these relationships based on experimental data and forecast data, we used the Search Tool for the Retrieval of Interacting Genes (STRING) database to identify interactions between the identified DE mRNAs (19). The Protein–Protein Interaction (PPI) network was visualized by Cytoscape in which the degree attribute of the DE mRNAs was >8 (20).

### Identification of Prognosis-Related Key mRNAs

The transcriptome sequencing data and corresponding clinical data of patients with LSCC were downloaded from TCGA database (up to April 07, 2020). The detailed TCGA IDs of LSCC patients including RNA-seq samples and miRNA-seq samples are shown in **Table S1**. Next, we performed univariate Cox proportional hazards analysis to obtain prognosis-related mRNAs based on TCGA LSCC cohort and a P-value <0.05 was considered statistically significant. Next, multivariate Cox regression analysis was performed using mRNAs with a P <0.05 and to remove potential confounding demographic factors including age and sex on the correlation between mRNAs and prognosis. We used the *R package ‘vioplot’* to generate a violin plot of the expression levels of key genes in LSCC samples. These relative expression levels were normalized and log2 transformed. Comparisons between the two groups (cancer tissues and adjacent non-cancer tissues) were made using non-parametric tests (Mann–Whitney test) as appropriate.

### Prediction of Key miRNA–mRNA Interactions

ENCORI was used to predict the targeted miRNAs for each of the eight key mRNAs; this is an open-source platform for studying mRNA–miRNA interactions and performing survival and differential expression analysis (21). The CLIP data filtering threshold was set to ‘three’ and the pan-cancer filtering threshold was set to ‘five’; these settings ensured high stringency when predicting the target miRNAs. Next, the data predicted by ENCORI and DE miRNAs analyzed for the TCGA LSCC cohort were combined and the overlaps were computed. We identified overlapping miRNAs as candidates for miRNA–mRNA interaction parings. Finally, the Kaplan-Meier survival analysis and correlation analysis conducted on these overlapping miRNAs with their target mRNAs identified prognostic related miRNA–mRNA pairing. The Kaplan–Meier method was used to generate survival curves for these patients with LSCC and to classify these patients into two groups according to gene expression (high or low). The median expression level was used as the cut-off point to classify patients into either the high expression group or the low expression group. Differences between the high and low expression groups were assessed using a log-rank test, and P <0.05 was regarded as statistically significant.

### Cell Culture and Transfection

HNSCC cell lines *Fadu*, *SCC-4*, *Cal-27*, and *Tu686* were purchased from the *Hunan Fenghui Biotechnology Co., Ltd*. *Fadu* and *Cal-27* cells were cultured in DMEM medium (BI, Israel) with 8% fetal bovine serum (FBS). *SCC-4* cells were cultured in RPMI-1640 medium (BI, Israel) with 8% FBS. *Tu686* cells were cultured in DMEM: F12 (1:1) medium (BI, Israel) with 8% FBS. All cells were cultured in medium supplemented with 1% PenStrep (100 U/ml penicillin and 100 μg/ml streptomycin) at 37°C in an atmosphere of 5% CO_2_. *SERPINE1* siRNA, *miR-181c-5p* mimic, and inhibitor (RiboBio, Guangzhou, China) were transfected using *riboFECT* (RiboBio, Guangzhou, China).

### Luciferase Reporter Assays

The 3′-UTR sequences of *SERPINE1* that including wild-type (WT) or mutant-type (Mut) *miR-181c-5p* binding sites were synthesized by Genecopoeia, Guangzhou, China. *Fadu* cells were co-transfected with *SERPINE1* 3′-UTR reporter plasmids [wild type (wt) or mutant (mut)] luciferase plasmids and *miR-181c-5p* mimic or mimic NC. After 48–72 h of transfection, luciferase activity was detected with a Cytation™ 5 system (BioTek, Winooski, VT, USA). *Renilla luciferase* activity was normalized to that of *Firefly luciferase* activity.

### Quantitative Real-Time PCR Analysis

Total RNA was extracted from HNSCC cells and homogenized in TRizol reagent (Life Technologies, Shanghai, China) in accordance with the manufacturer’s instructions. Reverse transcription was performed using a First Strand cDNA Synthesis Kit (Genecopeia, Guangzhou, China); qPCR assays were conducted using the All-in-One™ mRNA Detection kit (Genecopoeia, Guangzhou, China) based on SYBR-Green. MiRNA expression was measured using the All-in-One™ miRNA qRT-PCR detection kit (Genecopeia, Guangzhou, China). A QuantStudio7 Flex (Life Technologies, Carlsbad, CA) instrument was used to perform the qRT-PCR assay. Relative mRNA expression was normalized to *GAPDH* expression levels, and small RNA *RNU6* (*U6*) was used to normalize relative miRNA expression levels. The primers used in our analyses are shown in **Table 1**. Relative mRNA expression was measured using the 2−ΔΔCT method.

**Table 1:** Primers of RNAs for qRT-PCR validation.

Symbol	Primer sequence 5′–3′
SERPINE1	F: AATGGAAGTGAAGGAAGG
	R: AAAGTGCTGGGATTATAGG
E-cadherin	F: ATTGCAAATTCCTGCCATTC
	R: GTTGTCCCGGGTGTCATC
N-cadherin	F: CAGTGGCGGAGATCCTACTG
	R: CCTTGGCTAATGGCACTTG
Vimentin	F: TACAGGAAGCTGCTGGAAGG
	R: ACCAGAGGGAGTGAATCCAG
GAPDH	F: CAGGGCTGCTTTTAACTCTGG
	R: TGGGTGGAATCATATTGGAACA

### Cell Proliferation Assay

After transfection with *SERPINE1* siRNA, miRNA mimic, or miRNA inhibitor, cell proliferation rate was determined at 0, 24, 48, and 72 h using the Cell Counting Kit-8 (CCK8) (NCM Biotech, Suzhou, China); then the optical density (OD) value was measured at 450 nm on an enzyme-labeling instrument (BioTek, Winooski, VT, USA).

### Colony Formation Assay

Transfected *Fadu* or *SCC-4* were seeded in 6-well plates at 300 cells per well, then cultured for 14 days. Colonies were fixed by anhydrous methanol for 15 min, then left to get dry for 5 min at room temperature and stained with 0.2% crystal violet for 30 min. Images were obtained by digital capture and positive colonies was defined as those with >50 cells.

### Wound Healing and Cell Invasion Assay

Transfected *Fadu* or *SCC-4* cells were routinely cultured in 6-well plates until they reached monolayer confluence. Wounds were generated by scratching the monolayer of cells with a sterile 200-µl pipette tip; the cell debris was washed away with sterile 1× PBS. Images of the wound were captured at designated times (0, 48 h) to assess wound closure rates. The percent wound closure was calculated as: (1 − width at 48 h / width at 0 h) × 100%. Moreover, transwell assays were performed to explore cell invasion using a Matrigel invasion chamber (8 μm, BD Bioscience, USA). Briefly, cells (2 × 10^4^) were seeded in the upper chamber without serum. The lower chamber was filled with DMEM or RPMI-1640 medium with 8% FBS. Then cells were cultured for 48 h, and the invaded cells were stained with 0.2% crystal violet. All experiments were carried out in triplicate.

### Western Blot Assay

The total proteins were extracted from *Fadu* and *SCC-4* cells using RIPA lysis buffer (NCM Biotech, Suzhou, China), and the target proteins were detected by western blotting. Primary antibodies anti-PAI1 (A6211), anti-E-cadherin (A3044), anti-N-cadherin (A3055), and anti-Vimentin (A2584) were all purchased from Abclonal, Wuhan, China. Anti-GAPDH (AC001, Abclonal) was used to normalize the signals, and the second antibody, goat anti-rabbit IgG HRP-linked antibody, was purchased from Jackson Company (111-005-003, ImmunoResearch Laboratories, Inc. USA, 1:100,000).

### Statistical Analyses

All statistical data were analyzed using R version 3.6 or GraphPad Prism 8 (GraphPad Software, La Jolla, CA). Analysis of associations of gene expression levels in TCGA LSCC cohort were conducted using Student’s t test, while the Mann−Whitney U test was used to evaluate data with unequal variance. The log-rank test, Cox regression analysis and Kaplan−Meier method were used to evaluate associations between mRNAs, miRNAs and OS. A P-value <0.05 was regarded as statistically significant.

## Results

### Identification of DE mRNAs

**Figure 2:**
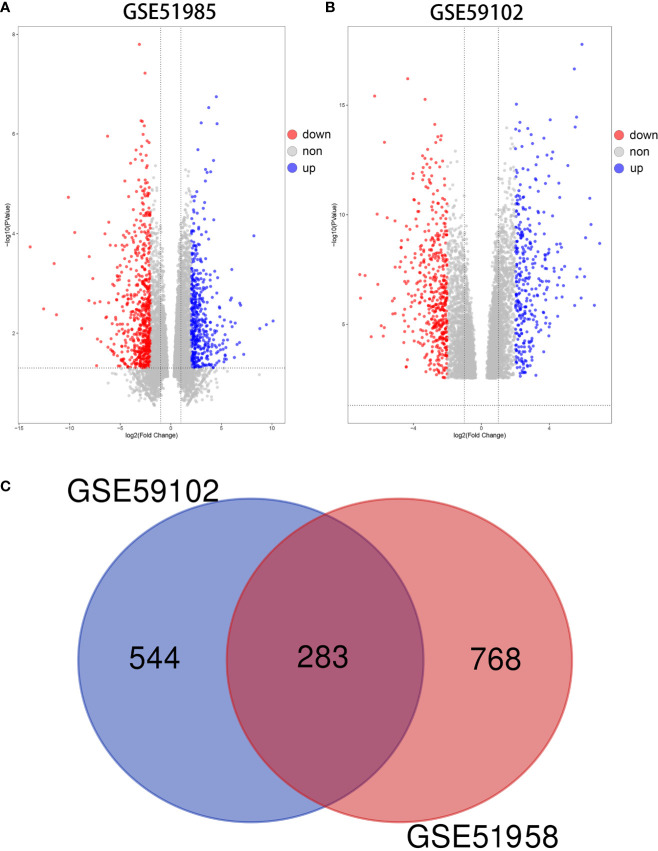
Differentially expressed mRNAs (DE mRNAs). **(A)** Volcano plot of DE mRNAs in GSE51985. **(B)** Volcano plot of DE mRNAs in GSE59102. **(C)** Venn diagram of 283 overlapping DE mRNAs.

**Figure 3:**
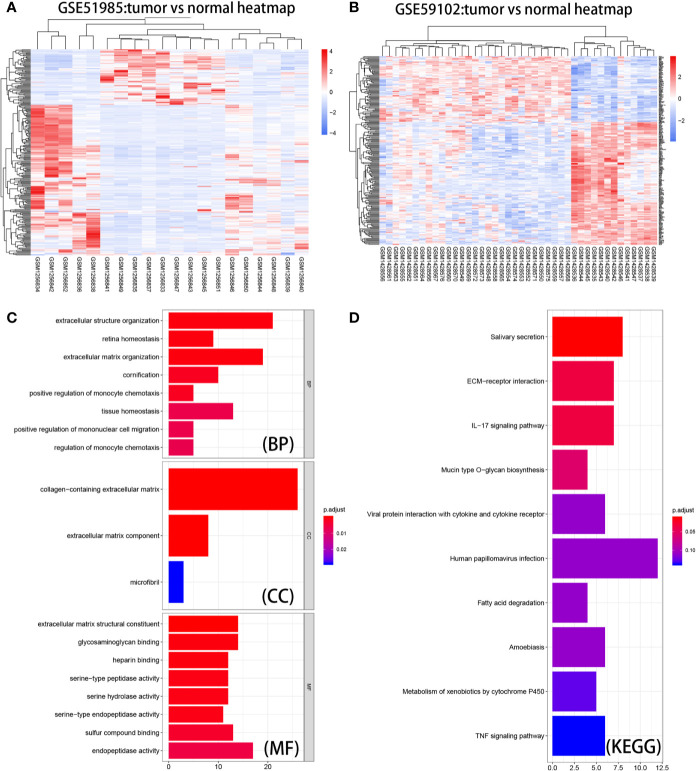
Heatmaps, GO enrichment, and KEGG pathway analysis of DE mRNAs. **(A)** Heat map of the overlapping DE mRNAs in GSE51985. **(B)** Heatmap of the overlapping DE mRNAs in GSE59102. **(C)** Biological process (BP), cellular component (CC), and molecular function (MF) of DE mRNAs. **(D)** KEGG pathway analysis of DE mRNAs.

Using the GEO2R algorithm, we identified a total of 1051 and 827 DE mRNAs from the GSE51985 and GSE59102 datasets, respectively (**Table S2**). A volcano plot was constructed by plotting the P-values of DE mRNAs *versus* the absolute log fold change (**Figures 2A, B****)**. Venn diagrams were constructed to identify a total of 283 overlapping genes (**Figure 2C**, **Table S2****)**. Two heatmaps for the mRNAs derived from the two datasets are shown in **Figures 3A, B**.

### GO Enrichment, KEGG Analysis, and PPI Network of DE mRNAs

**Figure 4:**
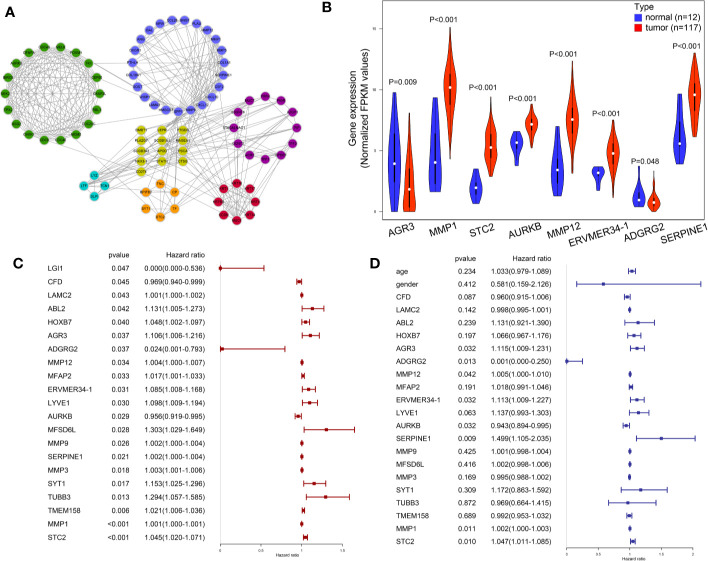
Visualization and identification of key mRNAs. **(A)** 84 candidate mRNAs’ PPI network. **(B)** Expression level of key mRNAs in cancer tissues and para-cancer normal tissues was compared in a violin plot. **(C)** Univariate Cox regression results of 21 candidate mRNAs in TCGA LSCC patients. **(D)** Multivariate Cox regression of eight key mRNAs in TCGA LSCC patients.

Based on results derived from the “clusterProfiler” package, we were able to gain a better understanding of the function of the DE mRNAs (**Figure 3C**). With regard to BP, the top ten enriched processes were extracellular structure organization, retina homeostasis, extracellular matrix organization, cornification, positive regulation of monocyte chemotaxis, tissue homeostasis, mononuclear cell migration, and regulation of monocyte chemotaxis. With regard to CC, the DE mRNAs showed enrichment in collagen-containing extracellular matrix, extracellular matrix component, and microfibrils. With regard to MF, the DE mRNAs were significantly enriched in extracellular matrix structural constituent, glycosaminoglycan binding, serine-type peptidase activity, serine hydrolase activity, serine-type endopeptidase activity, sulfur compound binding, and endopeptidase activity. As shown in **Figure 3D**, KEGG pathway enrichment analysis showed that the top three significantly enriched KEGG pathways were pathways associated with salivary secretion, ECM−receptor interaction, and the IL-17 signaling pathway. The information gained from these analyses could be used to explore the function of vital molecules during the development of LSCC. STRING analysis allowed us to build a PPI network for 283 DE mRNAs (**Figure S1****)**. We set ‘degree’ to above 8 as the cut-off criterion and subsequently visualized the interaction among 84 mRNAs (**Figure 4A**) using Cytoscape software.

### Selection and Expression Level of Key mRNAs

The LSCC RNA-Seq data of 123 samples (111 tumor, 12 normal), and their corresponding clinical information were retrieved and downloaded from TCGA database. The miRNA-Seq data of LSCC patients were obtained from 117 tumor samples and 12 normal samples. The potential effects of the 283 genes on the OS of TCGA LSCC patients were analyzed by univariate Cox regression analysis. A total of 21 mRNAs were significantly related to clinical outcome of LSCC patients (P < 0.05) (**Figure 4C**. Multivariate Cox regression analysis further indicated that eight mRNAs showed a significant prognostic value: *AGR3* [Hazard ratio (HR): 1.115 (1.009–1.231), P = 0.032], *ADGRG2* [HR: 0.001 (0.000–0.250), P = 0.013], *MMP12* [HR: 1.005 (1.000–1.010), P = 0.042], *ERVMER34-1* [HR: 1.113 (1.009–1.227), P = 0.032], *AURKB* [HR: 0.943 (0.894–0.995), P < 0.001], *SERPINE1* [HR: 1.499 (1.105–2.035), P = 0.009], *MMP1* [HR: 1.002 (1.000–1.003), P<0.011] and *STC2* [HR 1.047(1.011–1.085), P = 0.010] (**Figure 4D**). Moreover, the Mann−Whitney test revealed that the expression levels of six key mRNAs in LSCC were significantly higher than that of tissues adjacent to the tumors, whereas *AGR3* and *ADGRG2* were significantly expressed in lower levels in LSCC tissues compared to adjacent non-cancerous tissues (**Figure 4B**) (P < 0.05).

### Identification of *miR-181c-5p*/*SERPINE1* Signaling Pathway

**Figure 5:**
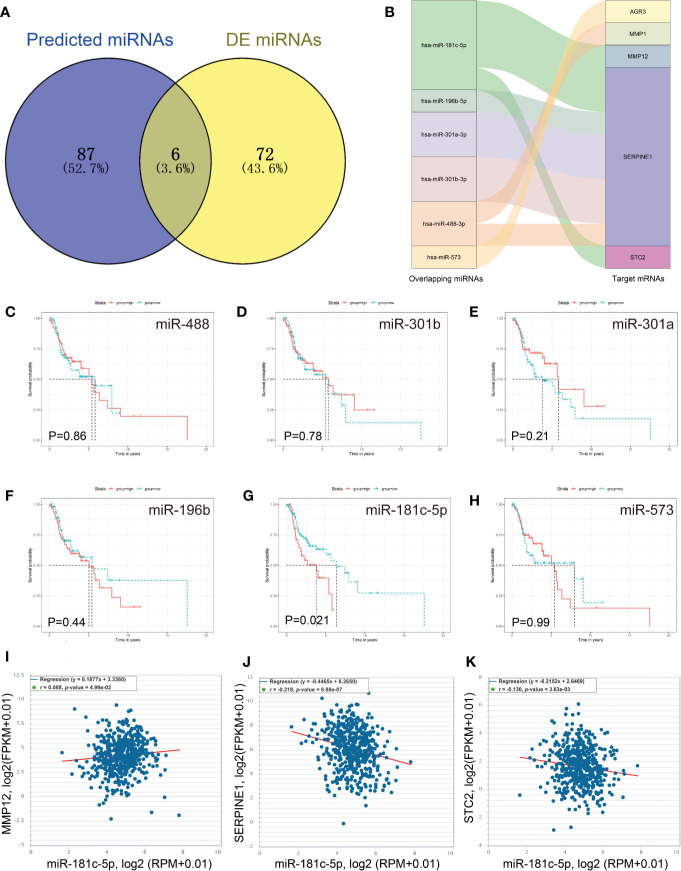
Identification of *miR-181c-5p* and *SERPINE1* interaction. **(A)** Predicted target miRNAs by overlapping two databases. **(B)** The key miRNA/mRNA interactions shown as a Sankey diagram. **(C–H)** Kaplan–Meier overall survival curve for patients according to miRNA expression. **(I**–**K)** The expression correlation of *miR-181c-5p* and *MMP12, SERPINE1 and STC2*.

It is known that miRNAs can suppress the expression levels of their target genes by acting as oncogenes or tumor suppressors, and thus play important roles in the regulation of cancer pathogenesis and metastasis (22). Therefore, we identified a total of 93 miRNAs strongly predicted to be associated with *AGR3*, *ADGRG2*, *MMP12*, *ERVMER34-1*, *AURKB*, *SERPINE1*, *MMP1* and *STC2* expression (**Figure 5A**). Of these, 78 DE miRNAs were filtered out from the TCGA LSCC cohort (**Table S3**). Six overlapping miRNAs, including *miR-181c-5p*, *miR-301a*, *miR-196b*, *miR-488*, *miR-301b*, and *miR-573* were identified as crucial target miRNAs of key mRNAs (**Figure 5B****)**. Among these six miRNAs, Kaplan−Meier survival analysis showed that only the low *miR-181c-5p* expression LSCC patient group exhibited poorer prognosis than the higher *miR-181c-5p* group (P = 0.021) (**Figures 5C–H**). Based on Spearman’s correlation analysis, a further inverse expression relationship between *miR-181c-5p* and *SERPINE1* was identified (correlation coefficient: −0.218; P = 8.88e-07) (**Figures 5I–K**).

### Overexpression of *miR-181c-5p* Inhibits Metastasis and EMT *In Vitro*

**Figure 6:**
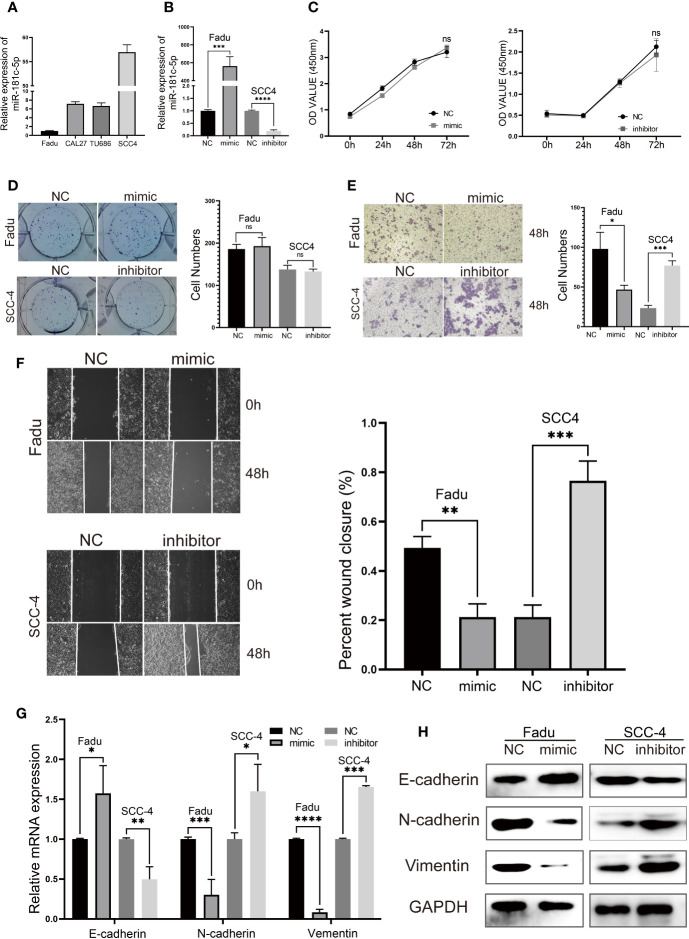
*miR-181c-5p* inhibits epithelial–mesenchymal transition (EMT) and metastasis in head and neck squamous cell carcinoma (HNSCC) cell lines. **(A)** qPCR results of 4 HNSCC cell lines showed the expression level of *miR-181c-5p*. **(B)** The expression of *miR-181c-5p* was successfully overexpressed or knocked down by *miR-181c-5p* mimic or inhibitor in *Fadu* and *SCC-4* cell lines. **(C)** The results of cell viability assay after cancer cells transfected *miR-181c-5p* mimic and inhibitor. **(D)** Colony formation assay were conducted in *Fadu* and *SCC-4* cells at the indicated time points. **(E)** Invasion of tumor cells through Matrigel in the transwell assay (magnification, ×100). **(F)** Wound healing assay revealed there was a different healing ratio between the group of negative control and mimic/inhibitor in *Fadu* and *SCC-4* cells following by transient transfected for 48 **(h) (G, H)** Expression of EMT markers including E-cadherin, N-cadherin, and vimentin in *Fadu* and *SCC-4* cells were detected by qRT-PCR and western blotting. ns, no significance; *P < 0.05; **P < 0.01; ***P < 0.001; ****P < 0.0001.

miRNAs have been proven to play important roles in HNSCC suppression or progression (23, 24). Initially, we tested whether *miR-181c-5p* mediated cell proliferation, migration, invasion, or EMT *in vitro*. Because laryngeal carcinoma cell line Hep2 has been largely contaminated by HeLa cell line, we used HNSCC cell lines for cell experiments. First, the expression of *miR-181c-5p* was quantified in four HNSCC cell lines (**Figure 6A**). We found that *miR-181c-5p* was expressed relatively higher in *SCC-4* cells but was expressed in lower levels in *Fadu* cells. Thus, *Fadu* and *SCC-4* cells were chosen to investigate the influence of *miR-181c-5p* on biological function. Next, we transfected the *miR-181c-5p* mimic and inhibitor into *Fadu* and *SCC-4* cells, respectively (**Figure 6B**) to determine their effects on biological function.

The cell viability assay and colony formation assay showed that *miR-181c-5p* had no effects on *Fadu* and *SCC-4* cell proliferation (**Figures 6C, D****)**. The transwell assay revealed a clear decrease in the invasive capacity of *Fadu* cells transfected with *miR-181c-5p* mimic (**Figure 6E**). In contrast, *SCC-4* cells transfected with inhibitor markedly promoted cell invasion when compared with the negative control transfection (**Figure 6E**). In addition, the wound healing assay indicated that overexpression of *miR-181c-5p* attenuated cell migratory capacity in *Fadu* cells, whereas this function was enhanced by silencing *miR-181c-5p* in *SCC-4* cells (**Figure 6F**). To further analyze the effects of *miR-181c-5p* on EMT in LSCC, the expression of E-cadherin, N-cadherin and vimentin were determined by qPCR (**Figure 6G**) and western blotting (**Figure 6H**). We found that overexpression of *miR-181c-5p* resulted in the up-regulation of E-cadherin and down-regulation of N-cadherin and vimentin in *Fadu* cells, while silencing *miR-181c-5p* induced repression of E-cadherin and enhancement of N-cadherin and vimentin in *SCC-4* cells. Consistent with these results, we revealed that *miR-181c-5p* inhibits cancer cells metastasis and EMT, indicating that the epithelial cells eliminated mesenchymal properties.

### *MiR-181c-5p* Restrains Migration and EMT Through the Regulation of *SERPINE1*

**Figure 7:**
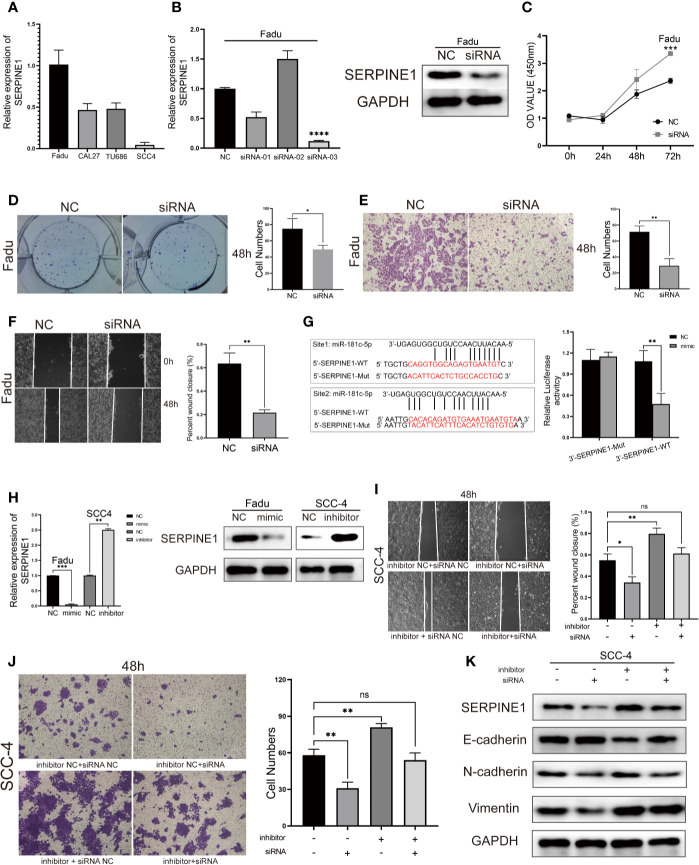
*SERPINE1* is a direct target of *miR-181c-5p* and rescues its inhibition in HNSCC cell lines. **(A)** qPCR results of four HNSCC cell lines showed the expression level of *SERPINE1*. **(B)** Expression level of SERPINE1 was significantly down-regulated after *SERPINE1* siRNA-03 transfection proved by qRT-PCR and western blotting. Effects of silencing *SERPINE1* on proliferation, migration, and metastasis in *Fadu* cells determined by CCK8 cell viability **(C)**, colony formation **(D)**, transwell migration **(E)** and wound-healing assay **(F)**. **(G)** Two binding sites of *miR-181c-5p* in *SERPINE1* 3′-UTR; mutations were designed as indicated, and luciferase reporter assays showed *SERPINE1* was a direct binding target of *miR-181c-5p*. Relative luciferase activity was expressed as firefly/Renilla luciferase activity. **(H)** Relative expression of *SERPINE1* mRNA and protein in *Fadu* cells that transfected with *miR-181c-5p* mimic, and in *SCC-4* cells transfected with *miR-181c-5p* inhibitor. Effects of restoration of *SERPINE1* on migration and metastasis in *SCC-4* cells determined by wound-healing **(I)**, transwell migration **(J)** and western blotting assay **(K)**. *P < 0.05; **P < 0.01; ***P < 0.001; ****P < 0.0001.

*SERPINE1* is considered an oncogene in various types of cancers and can enable cancer cells to gain new properties such as migration, EMT, and apoptosis resistance (25–26).The expression of *SERPINE1* was quantified in four HNSCC cell lines (**Figure 7A**). Then, in order to measure the potential effects of *SERPINE1* on LSCC, we transfected *SERPINE1* siRNAs to knockdown its expression in *Fadu* cells. Silencing of *SERPINE1* was confirmed by qPCR and western blotting (**Figure 7B**). The cell viability assay showed that silencing *SERPINE1* significantly inhibited *Fadu* cell proliferation at 72 h (**Figure 7C**). Furthermore, the colony formation ability of *Fadu* cells transfected siRNA was suppressed compared with that of the control group (**Figure 7D**). In addition, transwell assays (**Figure 7E**) and wound healing (**Figure 7F**) results demonstrated that *SERPINE1* knockdown slowed down migration and mitigated the invasion of *Fadu* cells. Altogether, these data suggest that the silencing of *SERPINE1*expression attenuates tumorigenicity and metastasis in HNSCC cells.

Next, we examined whether *miR-181c-5p* could target the regulation of *SERPINE1* expression. Two separate binding sites were predicted for the *miR-181c-5p* and *SERPINE1* mRNA sequence (**Figure 7G**). Luciferase reporter assays showed that transfection of *miR-181c-5p* mimic remarkably inhibited the luciferase activities of *SERPINE1*-3′UTR-WT compared with the negative control, whereas the mimic did not alter the luciferase activities of the *SERPINE1* 3′UTR-Mut (**Figure 7G**). *miR-181c-5p* mimic also significantly suppressed the expression of *SERPINE1* at both the mRNA and protein levels (**Figure 7H**), these findings were consistent with the results of the luciferase reporter assay. Next, we explored whether *miR-181c-5p* inhibited metastasis and EMT in function of *SERPINE1 expression*. Wound healing and transwell assays demonstrated that the repression of *miR-181c-5p* promoted migration and invasion in *SCC-4* cells, which was reversed by silencing of *SERPINE1* (**Figures 7I, J****)**. In addition, western blotting assays revealed that inhibition of *miR-181c-5p* decreased the protein expression of the epithelial marker E-cadherin, but increased mesenchymal markers N-cadherin and vimentin in *SCC-4* cells. Importantly, the effects of *miR-181c-5p* silencing were effectively reversed by *SERPINE1* siRNA co-transfection (**Figure 7K**). These results indicated that *miR-181c-5p* restrained cell migration and EMT through downregulation of *SERPINE1*.

## Discussion

The dysregulation of miRNAs has been reported to be involved in the tumorigenesis and progression of HNSCC, and to mediate cellular biological processes such as cell cycle regulation, differentiation, apoptosis, and migration in the epigenetic level (27–28). Several studies have reported expression profiles of miRNAs show remarkable diversity when comparing normal and malignant tissues, tumor types, as well as in different cancer stages, or prognosis (29, 30). In fact, specific miRNAs have found application as potential clinical diagnostic, prognostic, and therapeutic biomarkers in HNSCC (31). Studies have also revealed that miRNAs were dysregulated and associated with cellular proliferation, apoptosis and metastasis in LSCC cell lines, whereas most of these findings were based on *in vitro* experiments and have found limited application as biomarkers in the clinical setting (8, 32). In this study, using bioinformatics and RNA sequencing datasets from clinical tumor samples, we identified and validated the role of a miRNA which may potentially serve as a valuable candidate for diagnosis and therapeutics in LSCC.

We identified *eight* key mRNAs that associated with poor prognosis in patients with LSCC, as predicted by Cox regression and expression analysis. Among these, *MMP12*, *AURKB*, *SERPINE1*, and *MMP1* have been confirmed to play important roles in squamous cell carcinoma including LSCC (33–34). Currently, the influence of the other genes on LSCC has not been reported; however, they have been confirmed to be involved in several important signaling pathways in other human cancers. For example, *AGR3* has been shown to facilitate the stemness of colorectal cancer by regulating Wnt/*β*-catenin signaling (35); *STC2* has been shown to be involved in resistance to treatment with EGFR tyrosine kinase inhibitors and to promote the progression of lung cancer by regulating *JUN/AXL* signaling (36). Based on our expression and survival analysis results, we believe that the eight key mRNAs that we identified play a central part in the progression of LSCC.

The interplay between miRNA and target mRNA has achieved great interest in the field of tumor epigenetics. It has been extensively demonstrated that miRNAs act by inhibiting the expression of their targeted mRNAs (37). Our objective was to identify miRNAs having the potential ability to regulate the expression of key mRNA in LSCC. *MiR-181c-5p* was validated as the only candidate miRNA that not only was dysregulated in LSCC but its repressed expression was associated with poor clinical prognosis. *miR-181b-5p*, is a member of the miR-181 family, and has been reported to act as a tumor suppressor in a variety of tumors. miR-181b-5p targets the KPNA4 gene to inhibit EMT progression, and impede invasion and proliferation capacity of glioblastoma cells (38). In addition, Li et al. recently found that *miR-181c-5p* overexpression, through targeting of the *GSKIP* gene promotes E-cadherin expression in SiHa human cervical cancer cells, and repressed the expression of N-cadherin and vimentin (39) during EMT progression. Therefore, we boldly speculated that the dysregulation of this specific miRNA could result in abnormal biological functionality, including migration and invasion of LSCC cells.

Our *in vitro* experiments showed that miR-181b-5p is a key regulator of EMT and inhibits the cell invasion-metastasis cascade but not proliferation. Bioinformatics analysis demonstrated there was a negative correlation between miR-181b-5p and *SERPINE1* expression in LSCC, and the knockdown of *SERPINE1* attenuated tumor growth, EMT, and metastasis of cells, supporting its activity as an oncogene. Moreover, the downregulation of miR-181b-5p led to an elevated expression of *SERPINE1*, while up-regulation of miR-181b-5p resulted in the opposite tendency. These results revealed that miR-181b-5p repressed the mesenchymal phenotype in LSCC, at least in part, via directly targeting *SERPINE1*.

*SERPINE1*, a member of the urokinase plasminogen activating system (uPAS), was initially defined as a primary inhibitor of endogenous plasminogen activators and is synthesized in the liver and by fat tissue (40, 41). *SERPINE1* and its family members have been shown to promote different aspects of cancer development ranging from local proliferation to the migration and invasion of malignant cells (42, 43). Recent studies have also suggested that *SERPINE1* plays an important role in breast and pancreatic cancer (44, 45), and that the overexpression of *SERPINE1* could induce activation of the EGFR signaling pathway in breast cancer cells (44). Previous studies have shown that *SERPINE1* is abnormally expressed in HNSCC, whereas these studies did not attempt to identify the molecular mechanisms involved (40, 46). Herein, we demonstrated that the expression of *SERPINE1* was elevated in LSCC as a result of the down-regulation of miR-181b-5p and acts as one of the direct targets of miR-181b-5p. Further *SERPINE1* mediates the effects of miR-181b-5p on EMT and migration of LSCC cells.

Our findings revealed that *miR-181c-5p/SERPINE1* regulatory signaling was strongly associated with migration and invasion of LSCC, and *miR-181b-5p* acts as suppressor during the regulation of EMT progression. However, we acknowledge there are some limitations in this study. Further investigations focusing on clinical samples and the upstream signaling pathway and those of downstream of *miR-181c-5p*/*SERPINE1* in the development of LSCC are required.

## Conclusion

We used a series of integrated bioinformatics analyses to identify a new miRNA–mRNA signaling pathway for responsible in the pathogenetic mechanisms underlying LSCC. Based on our *in vitro* evidence we propose that *miR-181c-5p* might negatively regulate the expression of oncogene *SERPINE1*, and impede the invasion and metastasis by decreasing EMT. The components of the *miR-181c-5p/SERPINE1* signaling pathway may therefore be utilized as promising therapeutic targets and prognostic biomarkers in the future.

## Data Availability Statement

The original contributions presented in the study are included in the article/**Supplementary Materials**. Further inquiries can be directed to the corresponding author.

## Ethics Statement

The studies involving human participants were reviewed and approved by the Ethics Committee of Xiangya Hospital. The patients provided their written informed consent to participate in this study.

## Author Contributions

XL, WW, YL, XF, and YF performed the experiments, analyzed the data, and drafted the manuscript. PW, and YT designed the study and critically revised the manuscript. SZ polished the language. All authors contributed to the article and approved the submitted version.

## Funding

This research was supported by The National Natural Science Foundation of China (81302355).

## Conflict of Interest

The authors declare that the research was conducted in the absence of any commercial or financial relationships that could be construed as a potential conflict of interest.

## Abbreviations

LSCC, laryngeal squamous cell carcinoma; miRNAs, microRNAs; GEO, Gene Expression Omnibus; TCGA, The Cancer Genome Atlas; EMT, epithelial-to-mesenchymal transition; LC, laryngeal cancer; OS, overall survival; EMT-TF, epithelial-to-mesenchymal transition transcription factors; HNSCC, head and neck squamous cell carcinoma; DE miRNAs, differentially expressed miRNAs; DE mRNAs, differentially expressed mRNAs; FC, fold-change; GO, Gene Ontology; MF, molecular function; BP, biological process; CC, cellular component; STRING, Search Tool for the Retrieval of Interacting Genes; PPI, protein–protein interaction; FBS, fetal bovine serum; WT, wild-type; Mut, mutant-type; U6, small RNA RNU6; CCK8, Cell Counting Kit-8; OD, optical density; HR, hazard ratio.
