# Supplementary material for: Down-Regulation of MiR-181c-5p Promotes Epithelial-to-Mesenchymal Transition in Laryngeal Squamous Cell Carcinoma via Targeting SERPINE1
Source: Front Oncol. 2020 Dec 21;10:544476. doi: 10.3389/fonc.2020.544476 (PMC7931772; doi:10.3389/fonc.2020.544476)
Supplement: Supplementary file 1 [file DataSheet_1.docx]

Supplementary Material

# Supplementary Figures and Tables

## Supplementary Figures


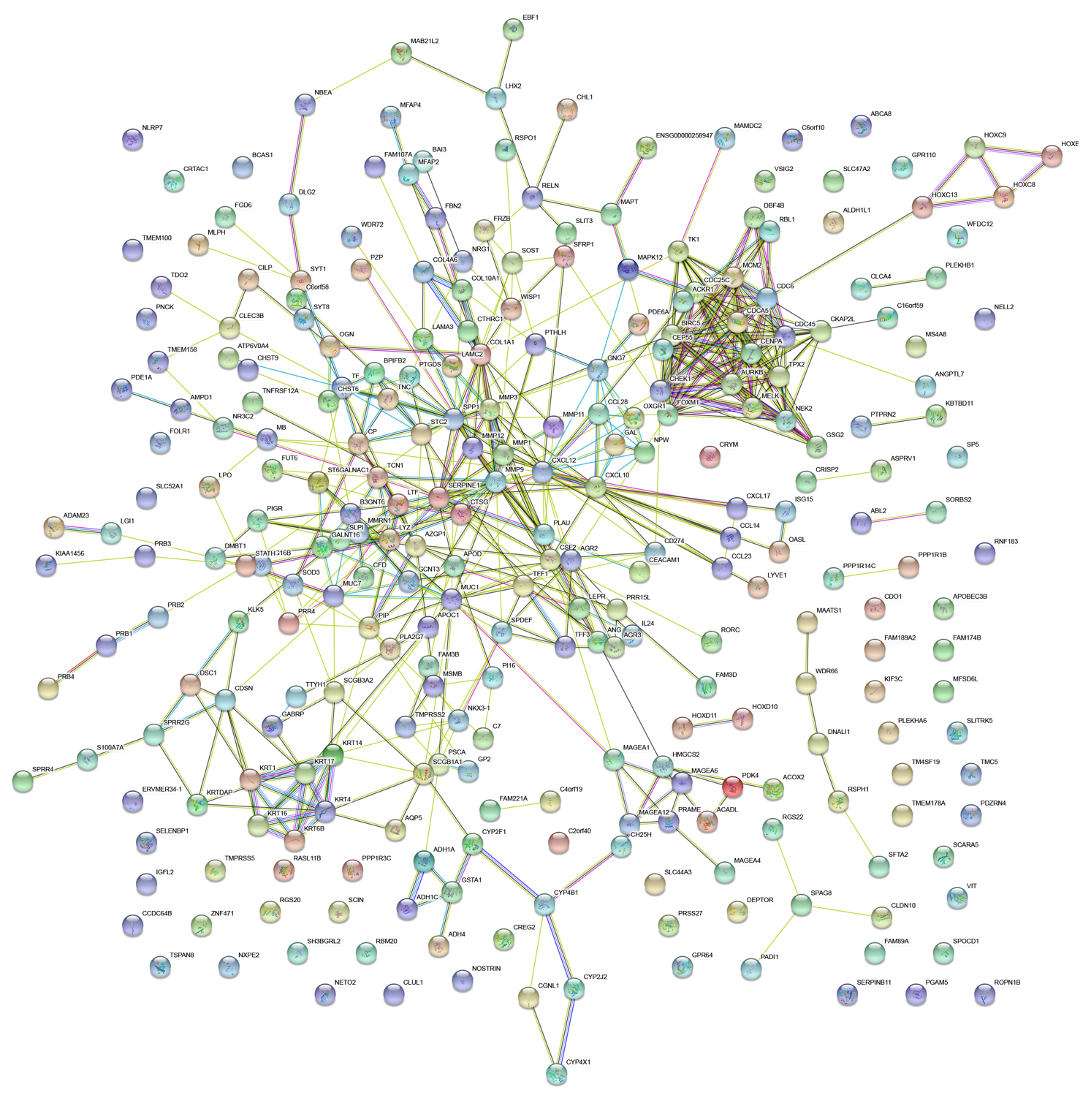


**Supplementary Figure 1.** The Protein-Protein Interaction network of 283 DE mRNAs constructed by STRING database.

## Supplementary Tables

**Supplementary Table 1.** TCGA IDs of RNA-seq and miRNA-seq samples.

**Supplementary Table 2.** mRNAs of GSE51985 and GSE59102 date sets and 283 overlapping mRNAs.

**Supplementary Table 3.** miRNAs predicted by ENCORI database and identified from TCGA LSCC cohort.
